# Survey of Medicinal Plants Used to Treat Malaria by Sidama People of Boricha District, Sidama Zone, South Region of Ethiopia

**DOI:** 10.1155/2016/9690164

**Published:** 2016-02-18

**Authors:** Solomon Asnake, Tilahun Teklehaymanot, Ariaya Hymete, Berhanu Erko, Mirutse Giday

**Affiliations:** ^1^Medicine and Health Science College, Hawassa University, P.O. Box 1560, Hawassa, Ethiopia; ^2^Aklilu Lemma Institute of Pathobiology, Addis Ababa University, P.O. Box 1176, Addis Ababa, Ethiopia; ^3^School of Pharmacy, Addis Ababa University, P.O. Box 1176, Addis Ababa, Ethiopia

## Abstract

In Ethiopia, malaria control has been complicated due to resistance of the parasite to the current drugs. Thus, new drugs are required against drug-resistant* Plasmodium* strains. Historically, many of the present antimalarial drugs were discovered from plants. This study was, therefore, conducted to document antimalarial plants utilized by Sidama people of Boricha District, Sidama Zone, South Region of Ethiopia. An ethnobotanical survey was carried out from September 2011 to February 2012. Data were collected through semistructured interview and field and market observations. Relative frequency of citation (RFC) was calculated and preference ranking exercises were conducted to estimate the importance of the reported medicinal plants in Boricha District. A total of 42 antimalarial plants belonging to 27 families were recorded in the study area. Leaf was the dominant plant part (59.0%) used in the preparation of remedies and oral (97.4%) was the major route of administration.* Ajuga integrifolia* scored the highest RFC value (0.80). The results of this study revealed the existence of rich knowledge on the use of medicinal plants in the study area to treat malaria. Thus, an attempt should be made to conserve and evaluate the claimed antimalarial medicinal plants with priority given to those that scored the highest RFC values.

## 1. Introduction

Malaria is a major public health problem in the tropical part of the world, especially in the Sub-Saharan Africa. It is estimated that annually there are 300 million cases of malaria worldwide resulting in one million deaths. Ninety percent of these deaths occur in Sub-Saharan Africa, and most of the victims are children under 5 years of age and pregnant women [1]. Malaria is a major obstacle to social-economic development in Africa. It accounts for 40.0% of public health expenditure, 10.0% of total disease burden, and 30.0%–50.0% of inpatient cases [2]. Furthermore, the disease affects children in their schooling and social development through both absence from school and permanent neurological or other types of damage associated with severe episodes.

Malaria is ranked as the leading communicable disease in Ethiopia; it is a leading cause of outpatient visits (17.0%), inpatient admissions (15.0%), and death (29.0%) in most parts of the country [3]. Chemotherapy is one of the control methods where different antimalarial drugs are widely used. However, due to the devastating nature of malaria and the failure of the most affordable drugs to treat the disease, there is still an urgent need to search for new and more effective antimalarial drugs. One of the approaches in the search for new antimalarial drugs is the use of traditional herbal remedies which have served as a source of the majority of conventional antimalarial drugs: quinine and artemisinin [4].

Different sociocultural groups in Africa possess detailed knowledge on the use of antimalarial plants that has been transferred from one generation to another usually through the word of mouth without proper documentation [5]. As a result, there is a danger of losing the knowledge due to passing away of knowledgeable people and the rapid degradation of natural habitats and ecosystems and thus there is a need for its documentation. In Ethiopia, although many plants have been claimed to have potential antimalarial properties, only few are documented and there are more that require documentation. Literature survey indicates that only two ethnobotanical surveys were conducted in two districts (Wondo Genet and Dale) of Sidama Zone, Southern Region of Ethiopia, to document the local uses of medicinal plants by the Sidama people, in which a total of eight antimalarial medicinal plants have been recorded [6, 7]. Thus, many more investigations need to be carried in Sidama Zone to come up with more complete information on medicinal plants of the Sidama people. Hence, the aim of the present study was to collect and document information on herbal remedies traditionally used for the treatment of malaria by the Sidama people of Boricha District of Sidama Zone in the South Nations, Nationalities, and Peoples Region (SNNPR) of Ethiopia. This study also aimed to investigate medicinal plant knowledge distribution among different social groups, marketability, and abundance of and threats to medicinal plants in the study area. The assessment of knowledge distribution among different social groups would help to identify groups with better knowledge of antimalarial plants in the study area for further and detailed investigations. The study was expected to come up with a list of antimalarial plants that could be further investigated and developed to be used as herbal remedies in primary healthcare system of the communities in the study area and elsewhere in the country. The information could also serve as a baseline data in the effort towards the development of conventional antimalarial drugs.

## 2. Materials and Methods

### 2.1. Description of the Study Area

**Figure 1:**
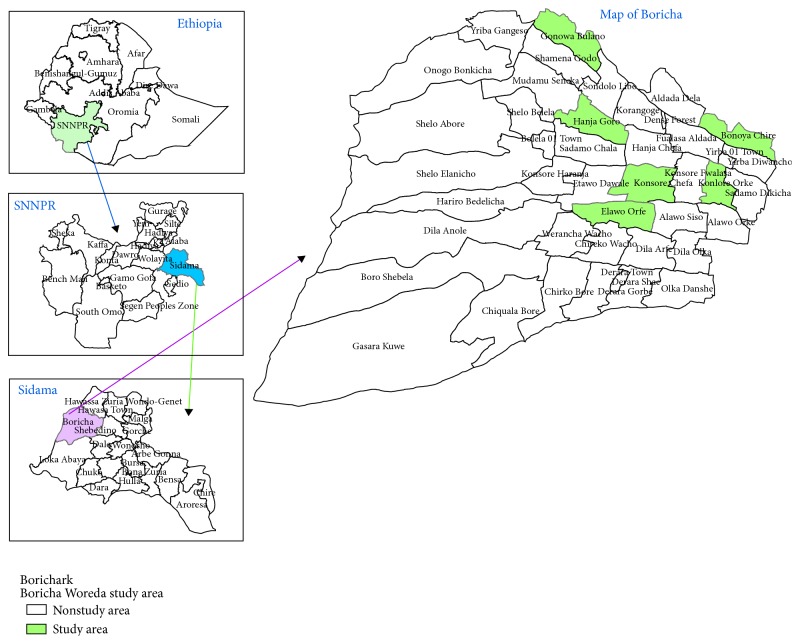
Map of Boricha District indicating study* kebeles*.

Boricha District is found in the Sidama Zone, SNNPR, Ethiopia. It is located between 6°46′N and 38°04′E and 7°01′N and 38°24′E. The district has an estimated area of 588.05 km^2^. It comprises 42* kebeles*: 39 are rural and three are suburban towns (Figure 1). The district is known to be malarious as altitude falls below 2000 m above sea level. Boricha has an estimated total population of 23,6341 of whom 118,566 are men and 117,775 are women. Close to 96.0% of the population is estimated to be rural inhabitants while about 4.0% are urban dwellers (Sidama Zone Finance and Economy Development Office, unpublished data of 2012). The majority of inhabitants in Boricha District belong to the Sidama ethnic group whose language (Sidama) belongs to the Cushitic language family. Land use in the district is dominated by rain-fed agriculture with small holding farms where cropping and dairy farming are commonly practiced. Maize, enset (*Ensete ventricosum*) coffee, chat (*Catha edulis*) potato, sugarcane, and sweet potato are the major products and are used for consumption and as a source of income (Boricha District Finance, Economy and Development Office, unpublished data of 2012). Although the vegetation in Boricha District and other surrounding localities has currently dwindled, the area was known to be covered by* Acacia* forest as recently as one generation ago [8].

There are 47 elementary schools and three high schools in Boricha District, in which a total of 57,098 students (53% males and 47% males) are attending their education (Boricha District Finance, Economy and Development Office, unpublished data of 2012). The district possesses six governmental health centers, one nongovernmental clinic, and thirty-nine health posts. In Boricha, the 10 leading diseases are malaria, intestinal parasites, diarrhea, upper respiratory tract diseases, urinary tract infection, rheumatism, skin diseases, fever of unknown diseases, eye diseases, and anemia (Boricha District Health Office, unpublished data of 2012).

### 2.2. Ethnobotanical Data Collection

The ethnobotanical survey was conducted between September 2011 and February 2012 and to document and analyze antimalarial plants used by communities in Boricha District. For the study, six* kebeles* were selected in consultation with Boricha District Administration, Health and Agriculture offices, and elderly people. Distribution of knowledgeable people and vegetation cover in the district were taken into consideration in the selection of the study* kebeles*. The selected* kebeles* were Boneya-Chire, Kpnsore-Chafe, Gonwa-Bulano, Alwa-Arfe, Hajnja-Goro, and Konsere-Arke with respective human populations of 4811, 6612, 8087, 5952, 6583, and 5201. Purposive sampling technique was employed in the selection of knowledgeable individuals belonging to the Sidama ethnic group from the six sampled* kebeles* in consultation with elderly people residing in the same* kebeles*. Ethnobotanical data were collected mainly through semistructured interview [9] conducted with 189 knowledgeable informants with age ranging from 20 years to 81 years, of which 124 (65%) were males and 65 (35%) were females. Of the total informants, 36 were from Kpnsore-Chafe, 33 from Hajnja-Goro, 32 each from Alwa-Arfe and Gonwa-Bulano, 30 from Konsere-Arke, and 26 from Boneya-Chire. The data gathered on antimalarial plants included plant local name, habit, part used, route of remedy administration, and information on their marketability and abundance/threat. Data were also collected through observation and guided field walks with informants. Information on marketability of antimalarial medicinal plants was gathered through a survey of four local markets (Balela, Konsore, Yirba, and Gulano) and interaction with plant sellers in the same markets. The interviews were conducted in Sidama language with the help of translators that grew up in the study area and having a good knowledge of the culture of the people.

### 2.3. Plant Specimen Collection and Identification

Voucher specimens of antimalarial medicinal plants were collected, dried, and identified using the different volumes of the Flora of Ethiopia and Eritrea. The specimens were further compared with authentic herbarium specimens and finally confirmed by specialists in the field and voucher specimens were deposited at the National Herbarium of Addis Ababa University.

### 2.4. Data Analysis

Data on informants' sociodemography and antimalarial plants used in Boricha District were entered into Excel spreadsheet. Knowledge on antimalarial plants among different social groups was compared using two-sample Wilcoxon rank sum (Mann-Whitney) test. A descriptive statistical procedure was employed to analyze data regarding plant habit, plant parts used and methods of preparation, dosages, and route of administration. Local importance of each plant species was determined by calculating relative frequency of citation (RFC) [10], using the formula, RFC = FC/*N*, where FC is the number of informants who mentioned the use of the species and* N* is the total number of informants. Preference ranking exercise was conducted with 10 informants, randomly selected from among the 189 knowledgeable informants that had participated in the interviews, to rank seven most frequently cited medicinal plants used to treat malaria based on interview results following the approach of Martin [9].

### 2.5. Ethical Considerations

Prior to conducting the current study, approval letter was received from Institutional Review Board of Aklilu Lemma Institute of Pathobiology, Addis Ababa University, and verbal informed consent was obtained from each informant who participated in the study following explanation on the purpose of the study. Permission to conduct the study was obtained from Boricha District Administration and community leaders.

## 3. Results

### 3.1. Comparison of Knowledge among Different Social Groups

A total of 189 informants took part in the ethnobotanical investigation to document medicinal plants used to manage malaria locally known as shekere. Although more medicinal plants were reported by males than females, there was no significant difference (*P* > 0.05) between mean numbers of antimalarial plants reported. On the other hand, there were significant differences in the mean numbers of reported antimalarial plants between two age groups and two groups of different literacy levels (*P* < 0.05). The mean number of antimalarial plants reported by elders was higher than that reported by young informants. Similarly, the mean number of antimalarial plants reported by illiterates was higher than that reported by literates (Table 1).

### 3.2. Antimalarial Medicinal Plants

The ethnobotanical study revealed a total of forty-two antimalarial plant species belonging to 39 genera and 27 plant families. The family Lamiaceae contributed the highest number of antimalarial plants (7 species) followed by Asteraceae (3 species). The families Fabaceae, Brassicaceae, Rutaceae, Cucurbitaceae, Euphorbiaceae, Solanaceae, and Ranunculaceae were represented by 2 species each, while 18 families were represented by single species each. Of the total antimalarial plants, 39 were identified to species level and two were identified to a genus level. The species name, family and vernacular names, habit, plant part used, method of preparation, and voucher specimen numbers are presented in Table 2.

### 3.3. Growth Forms of the Antimalarial Medicinal Plants and Their Habitat

Shrubs and herbs are the commonly used growth forms comprising 39.0% and 30.0% of the antimalarial plants, respectively, followed by trees (16.0%) and climbers (15.0%). Interview results indicated that most (79.6%) of the antimalarial medicinal plants in the study area were uncultivated ones while 15.4% were cultivated in homegardens and 5.0% were collected from both the wild and homegardens. The antimalarial plants were found to be primarily cultivated for their uses as food, spice, shade, and animal feed. Most of the uncultivated antimalarial medicinal plants were harvested from forests (49.0%) and roadsides (39.0%).

### 3.4. Part Used and Condition of the Antimalarial Medicinal Plants

Leaf was the most frequently used plant part (59.0%) for the preparation of the remedies, followed by aerial part (12.0%), twig and root (10.0% each), and seed (7.0%). The majority (89.1%) of the antimalarial plants were prepared from fresh plant parts and 10.9% were used in their dried forms. The informants also reported that the parts were collected when needed, and there was no specific time needed for their collection. It was also reported that rituals were not performed during collection or processing of herbal remedies.

### 3.5. Method of Preparation, Route of Administration, and Dosage

Pastes or powders were usually by using mortar and pestle locally made of wood and grinder made of stone. Decoction (49.0%) and homogenization (37.0%) were the two commonly used methods in the preparation of remedies, followed by chewing (7.0%), concoction (5.0%), and steam bath (2.0%). The majority of the antimalarial plants were prepared and administered without the use of diluents. Almost all remedies were taken orally (97.6%). Lack of precision in the preparation of remedies was frequently reported.

In most cases, the prepared remedies were given once a day and treatments were completed within 3 days. In few cases, treatments could be extended beyond 3 days. Tea or coffee cup was frequently used to measure dose of remedies administered orally. Dosage administered to patients differed based on age where children (below 18 years of age) were given 1/2 or 1/3 of the dose given to adults. According to the informants, the claimed antimalarial remedies had no side effects and hence no antidotes were mentioned. Coffee with some amount of salt was used as an additive for some of the remedies. Most of the informants believed that patients get cured of malaria after treatment with plant remedies was completed. If patients did not show signs of improvement, they were advised to go to a nearby health post or health center.

### 3.6. Marketability of Antimalarial Medicinal Plants

Most informants mentioned that the majority of the remedies (85.8%) were not sold at local markets. Parts of the remedies were rather harvested, processed, and used whenever required. Only few antimalarial medicinal plants (12.0%) were reported to be sold in local markets primarily for other uses, which were also noted during visits made to different local markets (Table 3).

### 3.7. Abundance of and Threats to Antimalarial Medicinal Plants

Informants also mentioned that most of the antimalarial medicinal plants were not easily found in nearby areas and collectors had to travel long distances to harvest them. Of the antimalarial plants, 9.5% were reported as abundant, 54.8% as rare, and 35.7% as very rare. Different factors were mentioned by the informants as main threats for medicinal plants. Deforestation was reported by 50.0% of the informants as the main threat for depletion of the medicinal plants in the study area, followed by agricultural expansion (35.0%), shortage of rain (12.0%), and charcoal and firewood trading (10.0%). According to informants, few efforts have so far been made in the study district to conserve medicinal plants.

### 3.8. Estimation of the Importance of Antimalarial Plants

Relative frequency of citation (RFC) values were calculated to get an estimation of the importance of certain antimalarial plants in the study area. Accordingly,* Ajuga integrifolia* (RFC = 0.80),* Peponium vogelii* (RFC = 0.75),* Melia azedarach* (RFC = 0.68),* Premna schimperi* (RFC = 0.63), and* Clerodendrum myricoides* (RFC = 0.60) were found to score the highest RCF values (Table 2).

Preference ranking exercises conducted on seven antimalarial plants resulted in* Ajuga integrifolia* getting the highest score, followed by* Melia azedarach*,* Peponium vogelii*,* Premna schimperi*,* Clerodendrum myricoides*,* Croton macrostachyus*, and* Fagaropsis angolensis* (Table 4).

## 4. Discussion

The report of a high number of antimalarial plants (42 species) from the study area indicates the importance of the medicinal flora of the study area in the day-to-day management of malaria. Lamiaceae and Asteraceae were found to contribute the highest number of species, followed by families Rutaceae, Cucurbitaceae, Euphorbiaceae, and Fabaceae. An ethnobotanical investigation carried out in Eastern Ethiopia also revealed the better contribution of antimalarial plants by the families Fabaceae, Euphorbiaceae and Lamiaceae, and Asteraceae [11]. Asteraceae and Lamiaceae are among the largest dicotyledonous families in the Flora of Ethiopia and Eritrea containing about 440 and 170 species, respectively [12, 13]. The fact that the two families are relatively rich in medicinal plant species might also indicate their richness in some active principles. Literature survey indicates that 19 (45.0%) of the antimalarial plant species compiled during the current investigation are also used for the same purpose elsewhere in Ethiopia [11–27]. The literature survey further revealed that three (*Melia azedarach*,* Allium sativum*, and* Vernonia amygdalina*) of the currently documented antimalarial plants are also used to treat the same disease by the Sidama people residing in other districts of the Sidama Zone [6, 7], which might indicate their better efficacy in the treatment of malaria. Antimalarial plants mentioned three or more times in studies conducted elsewhere in the country included* Allium sativum* [6, 7, 14, 16, 19, 20, 25, 26, 28–32],* Aloe* sp. [11, 15, 16],* Brucea antidysenterica* [22, 23, 33, 34],* Calpurnia aurea* [14, 16, 21, 23],* Clerodendrum myricoides* [16, 17, 21, 23, 35],* Croton macrostachyus* [14, 20, 21, 23, 24, 26, 29, 32, 35, 36],* Dodonaea angustifolia* [14, 16, 21, 27],* Lepidium sativum* [14, 20, 25, 29, 34, 37],* Melia azedarach* [6, 18, 20, 31], and* Vernonia amygdalina* [6, 7, 20, 23, 24, 38, 39]. According to Trotter and Logan, plants which are used in some repetitive fashion are more likely to be biologically active [40]. Few (*Aloe* sp.,* Clerodendrum myricoides*,* Croton macrostachyus*,* Dodonaea angustifolia*,* Solanum incanum*, and* Vernonia amygdalina*) of the currently reported antimalarial plants have been evaluated for their antiplasmodial properties [11, 41–44] and showed activity.

The study indicated that no significance difference was observed between the mean numbers of medicinal plants reported by males and females, which is in agreement with result reported elsewhere [30, 45]. On the other hand, significance difference was noted between the mean numbers of medicinal plants reported by two age groups (elders and young) and that of informants of two different literacy levels (*P* < 0.05). This could be attributed to the less attention given by the young towards traditional medical system due to the influence of modernization related to urbanization, formal education, and less effort of knowledge owners to systematically transfer their knowledge to others. Similar results were reported in studies conducted in other parts of Ethiopia [30, 46–48], India [49], and Brazil [50].

Most of the antimalarial medicinal plants were harvested from the wild. Only few species were harvested from cultivation areas. Ethnobotanical investigations carried in different parts of Ethiopia [11, 48, 51], Ghana [52], and Kenya [53] indicate that most medicinal plants are uncultivated ones and are obtained from the wild. Such fact indicates the high level of threat to medicinal plants in the study area amid ongoing deforestation and habitat destruction.

Shrubs were the most commonly used plant forms used by the Sidama people of Boricha District in the preparation of antimalarial remedies, followed by herbs and trees. Elsewhere in Ethiopia [46, 54], similar results were reported where shrubs are the commonly used antimalarial plants, followed by herbs. Our result is also consistenct with that of a study conducted in Kenya where shrubs are the most frequently used growth form, followed by herbs [53]. The frequent use of shrubs might be related to their better availability in both the dry and wet seasons as compared to other growth forms. However, ethnobotanical investigations carried out in Tehuledere District, South Wollo [55], and southwest Ethiopia [56] indicate the frequent use of herbaceous species.

Leaf was the most reported plant part for the preparation of antimalarial remedies in the study area. This is in agreement with results of studies conducted in other parts of Ethiopia [48, 55]. Studies carried out in other parts of Africa have also reported similar findings [52, 53, 57]. The preference of leaves to other plant parts could be related to their easy accessibility and simplicity in remedy preparation or presence of high amount of active constituents. The preference for leaves could be due to the less impact of harvesting such part on individual plants as compared to roots, stems bark, and flowers [51].

Most of the antimalarial remedies in Boricha District were prepared from a single plant species, which is in agreement with studies carried out elsewhere in Ethiopia [11] and Kenya [53]. On the other hand, a study conducted in Sekoru District of Ethiopia reported the common use of mixtures of different plant species in the preparation of remedies [54]. The frequent use of concoctions could be related to the belief of synergic reactions where one plant could have a potentiating effect over the other [58]. Decoction was the most frequently used method of remedy preparation in the study area, which is in agreement with results of studies conducted in Kenya [53, 59].

The majority of antimalarial remedies were prepared from fresh materials. Investigations conducted elsewhere also indicated the wider use of fresh plant materials in remedy preparation [60, 61]. Storage of remedies was not commonly reported by the informants, which might be attributed to the year-round availability of medicinal plant species most of which were reported to be woody species. Moreover, the common use of fresh plant materials might also be taken as an attempt not to lose volatile substances that contribute to the efficacy of the remedies [62].

Analysis of interviews and local market surveys in Boricha District revealed that few medicinal plants were sold in the local markets but primarily for other uses as users could easily and freely harvest them from their immediate environment. Our finding is in agreement with those reported elsewhere in Ethiopia [48, 54, 63]. Preference ranking exercises conducted on seven medicinal plants that scored the highest RFC values for their use against malaria revealed* Ajuga integrifolia* as the most preferred plant, which might indicate its better effectiveness in the treatment of malaria.

## 5. Conclusions

The study indicates that the Sidama people of Boricha District have rich traditional knowledge concerning the use of antimalarial medicinal plants. Such traditional knowledge has continued to be important for the community and is still catering for their primary health care. It was also revealed that relatively older people have better knowledge on the use of medicinal plants as compared with younger ones indicating that the latter have less interests towards the traditional medical system. For the practice to continue or sustain, there is a need to develop a strategy which would help to conserve and promote medicinal plant resources in the study area such as growing medicinal plants in homegardens and setting aside a reserved area for their protection. The safety and efficacy of the reported antimalarial plants need to be evaluated, with priority given to those antimalarial plants with highest informant consensus, before they are recommended for wider use and further pharmacological and phytochemical investigation.

## Figures and Tables

**Table 1:** Comparison of the mean numbers of reported antimalarial plants between different social groups of the Sidama people of Boricha District, South Region of Ethiopia.

Social group	Respondent type	Number of respondents	Average number of antimalarial plants (mean ± SEM)	Median (IQR)	*P* value
Gender	Male	119	5.2 ± 0.34	4.0 (3.0–6.0)	0.096
	Female	70	4.7 ± 0.21	4.5 (4.0–5.0)	

Age	Young (20–39 years)	67	3.3 ± 1.30	3.0 (2.0–4.0)	<0.001
	Elders (≥40 years)	122	5.8 ± 2.58	5.0 (4.0–7.0)	

Literacy level	Illiterate	148	5.1 ± 0.21	4.0 (3.5–6.0)	<0.002
	Literate	41	4.0 ± 0.33	3.0 (3.0–5.0)	

IQR: interquartile range (25% percentile–75% percentile).

**Table 2:** Antimalarial medicinal plants of the Sidama people of Boricha District, South Region of Ethiopia, alphabetically arranged by family and scientific names.

Family	Scientific name	Sidama name	Habit	Method of preparation	Route of administration	Part used	Voucher number	RFC
Acanthaceae	*Hypoestes forskaolii* (Vahl) R.Br.	Chikecho	Herb	Homogenization	Oral	Leaf	BOR 24	0.010
Alliaceae	*Allium sativum* L.	Tuma	Herb	Chewing, pounding	Oral	Stem	BOR 42	0.080
Aloaceae	*Aloe *sp.	Argessa	Shrub	Homogenization	Oral	Leaf	BOR 2	0.040
Apocynaceae	*Carissa spinarum *L.	Kirketcho	Herb	Homogenization	Oral	Root	BOR 19	0.011
Asteraceae	*Lactuca glandulifera* Hook.f.	Maracha	Herb	Infusion	Oral	Aerial	BOR 14	0.011
	*Vernonia amygdalina *Del.	Hecho	Shrub	Homogenization	Oral	Leaf	BOR 11	0.110
	*Vernonia auriculifera *Hiern	Regicho	Shrub	Decoction	Oral	Leaf	BOR 26	0.021
Boraginaceae	*Cynoglossum coeruleum *Hochst	Batartusa	Herb	Infusion	Oral	Aerial	BOR 31	0.032
Brassicaceae	*Brassica oleracea *L.	Shena	Shrub	Homogenization	Oral	Seed	BOR 3	0.030
	*Lepidium sativum *L.	Heto	Herb	Homogenization	Oral	Seed	BOR 34	0.011
Celastraceae	*Maytenus arbutifolia *(A. Rich.) Wilc	Borbodich	Shrub	Chewing	Oral	Root	BOR 36	0.021
Cucurbitaceae	*Cucumis dipsaceus *Ehrenb. ex Spach	Basu-bakule	Climber	Infusion	Oral	Leaf	BOR 6	0.026
	*Peponium vogelii *(Hook.f.) Engl.	Surupa	Climber	Decoction	Oral	Leaf	BOR 10	0.750
Euphorbiaceae	*Croton macrostachyus *Del.	Masincho	Tree	Decoction	Oral	Leaf	BOR 7	0.130
	*Euphorbia schimperiana *Scheele	Bingele	Shrub	Homogenization	Oral	Leaf	BOR 17	0.011
Fabaceae	*Albizia schimperiana *Oliv.	Maticho	Shrub	Decoction	Oral	Leaf	BOR 39	0.070
	*Calpurnia aurea* (Ait.) Benth.	Chekata	Shrub	Decoction	Oral	Leaf	BOR 41	0.011
Lamiaceae	*Ajuga integrifolia *Buch.-Ham. ex D.Don	Anamuro	Herb	Decoction	Oral	Aerial	BOR 9	0.800
	*Clerodendrum myricoides *(Hochst.)	Madesisa	Shrub	Infusion	Oral	Leaf	BOR 32	0.600
	*Leucas *sp.	Blbalate	Herb	Homogenization	Oral	Aerial	BOR 15	0.037
	*Ocimum lamiifolium *Hochst ex. Benth.	Chabicha	Shrub	Concoction	Nasal	Twigs	BOR 8	0.063
	*Ocimum urticifolium *Roth.	Angabisha	Shrub	Concoction	Nasal	Leaf	BOR 1	0.063
	*Premna schimperi *Engl.	Udo	Shrub	Homogenization	Oral	Leaf	BOR 18	0.630
	*Satureja punctata *(Benth.) Briq.	Amessa	Herb	Homogenization	Oral	Aerial	BOR 22	0.048
Loganiaceae	*Nuxia congesta *R.Br. ex Fresen.	Nole	Shrub	Infusion	Oral	Leaf	BOR 33	0.021
Meliaceae	*Melia azedarach *L.	Mime	Tree	Homogenization	Oral	Twigs	BOR 5	0.680
Melanthiaceae	*Bersama abyssinica *Fresen.	Oloncho	Shrub	Homogenization	Oral	Leaf	BOR 21	0.021
Menispermaceae	*Stephania abyssinica *(Dillon & A. Rich)	Kalala	Climber	Infusion	Oral	Root	BOR 27	0.026
Moringaceae	*Moringa stenopetala * (Bak.f.) Cuf.	Shifera	Tree	Homogenization	Oral	Leaf	BOR 13	0.030
Myrtaceae	*Eucalyptus globulus *Labill	Wago-barzafe	Tree	Steam bath	Whole body	Leaf	BOR 29	0.032
Papaveraceae	*Argemone mexicana *L.	Kolcolich	Shrub	Decoction	Oral	Root	BOR 23	0.026
Ranunculaceae	*Clematis hirsuta* Perr. & Guill.	Fede	Herb	Homogenization	Oral	Leaf	BOR 25	0.021
	*Clematis simensis *Fresen	Sido	Climber	Decoction	Oral	Leaf	BOR 40	0.021
Rubiaceae	*Rubia cordifolia *L.	Hare	Climber	Chewing	Oral	Root	BOR 37	0.021
Rutaceae	*Fagaropsis anolensis *(Engl.) Dale	Godecho	Tree	Chewing	Oral	Seed	BOR 4	0.130
	*Ruta chalepensis *L.	Sunkuruta	Shrub	Decoction	Oral	Twigs	BOR 12	0.048
Santalaceae	*Osyris quadripartita *Decn.	Karicho	Shrub	Infusion	Oral	Leaf	BOR 38	0.016
Sapindaceae	*Dodonaea angustifolia *L.f.	Entalcha	Shrub	Decoction	Oral	Leaf	BOR 20	0.011
Simaroubaceae	*Brucea antidysenterica *J.F.Mill.	Hatawicho	Tree	Infusion	Oral	Leaf	BOR 30	0.020
Solanaceae	*Solanum incanum *L.	Chucho	Shrub	Infusion	Oral	Leaf	BOR 35	0.011
	*Withania somnifera *(L.) Dunal	Gizawicho	Shrub	Homogenization	Oral	Leaf	BOR 16	0.074
Vitaceae	*Cyphostemma niveum *(Hochst. ex Schweinf.)	Dashe	Climber	Infusion	Oral	Leaf	BOR 28	0.040

**Table 3:** Antimalarial medicinal plants of the Sidama people of Boricha District, South Region of Ethiopia, sold in local markets.

Scientific name	Local name	Primary purpose
*Allium sativum*	Tuma	Food
*Brassica oleracea*	Shena	Food
*Cucumis dipsaceus*	Basu-bakule	Veterinary medicine
*Eucalyptus globulus*	Wago-barzafe	Construction material, firewood
*Lepidium sativum*	Heto	Spice
*Ruta chalepensis*	Sunkuruta	Spice

**Table 4:** Preference ranking of seven medicinal plants used to treat malaria by the Sidama people of Boricha District, South Region of Ethiopia.

Antimalarial plants	Participants labeled A–J										Total score	Rank
	A	B	C	D	E	F	G	H	I	J		
*Ajuga integrifolia*	6	7	3	7	4	5	7	4	6	7	56	1
*Melia azedarach*	7	6	5	3	6	6	6	3	2	5	50	2
*Peponium vogelii*	5	1	4	1	7	7	3	5	7	6	46	3
*Premna schimperi*	4	5	6	6	5	1	2	7	3	3	42	4
*Clerodendrum myricoides*	3	2	7	5	2	4	1	6	5	1	37	5
*Croton macrostachyus*	1	4	2	2	3	2	4	2	4	2	27	6
*Fagaropsis angolensis*	2	3	1	4	1	3	5	1	1	4	25	7

## References

[1] WHO (2015). *World Malaria Report*.

[2] WHO/UNICEF (2003). *The African Malaria Report 2003*.

[3] CSA (2006). *The Ethiopian Demographic and Health Survey*.

[4] Chen C. (2014). Development of antimalarial drugs and their application in China: a historical review. *Infectious Diseases of Poverty*.

[5] Asase A., Kokubun T., Grayer R. J. (2008). Chemical constituents and antimicrobial activity of medicinal plants from Ghana: *Cassia sieberiana*, *Haematostaphis barteri*, *Mitragyna inermis* and *Pseudocedrela kotschyi*. *Phytotherapy Research*.

[6] Tamene S. (2011). *An ethnobotanical study of medicinal plants in Wondo genet natural forest and adjacent kebeles, Sidama Zone, SNNP Region, Ethiopia [M.S. thesis]*.

[7] Kewessa G., Abebe T., Demissie A. (2015). Indigenous knowledge on use and management of medicinal trees and shrubs in Dale District. *Ethnobotany Research & Applications*.

[8] USAID (2005). *Ethiopia Southern Nations, Nationalities and Peoples Region (SNNPR) Livelihood Zone Reports: SNNPR Follow-On to Regional Livelihoods Baseline Study*.

[9] Martin G. J. (1995). *Ethnobotany: A Method Manual*.

[10] Tardío J., Pardo-De-Santayana M. (2008). Cultural importance indices: a comparative analysis based on the useful wild plants of Southern Cantabria (Northern Spain). *Economic Botany*.

[11] Mesfin A., Giday M., Animut A., Teklehaymanot T. (2012). Ethnobotanical study of antimalarial plants in Shinile District, Somali Region, Ethiopia, and *in vivo* evaluation of selected ones against *P. berghei*. *Journal of Ethnopharmacology*.

[12] Tadesse M. (2004). *Flora of Ethiopia. Volume 4, Part 2: Asteraceae (Compositae)*.

[13] Ryding O., Hedberg I., Kelbessa E., Edwards S., Demissew S., Persson E. (2006). Lamiaceae. *Flora of Ethiopia and Eritrea. Volume 5: Gentianaceae to Cyclocheilaceae*.

[14] Berhanu A., Asfaw Z., Kelbessa E. (2006). Ethnobotany of plants used as insecticides, repellents and anti-malarial agents in Jabitehnan District, West Gojjam. *SINET: Ethiopian Journal of Science*.

[15] Seifu T., Asres K., Gebre-Mariam T. (2006). Ethnobotanical and ethnopharmaceutical studies on medicinal plants of Chifra District, Afar Region, North Eastern Ethiopia. *Ethiopian Pharmaceutical Journal*.

[16] Giday M., Teklehaymanot T., Animut A., Mekonnen Y. (2007). Medicinal plants of the Shinasha, Agew-awi and Amhara peoples in northwest Ethiopia. *Journal of Ethnopharmacology*.

[17] Wondimu T., Asfaw Z., Kelbessa E. (2007). Ethnobotanical study of medicinal plants around 'Dheeraa' town, Arsi Zone, Ethiopia. *Journal of Ethnopharmacology*.

[18] Flatie T., Gedif T., Asres K., Gebre-Mariam T. (2009). Ethnomedical survey of Berta ethnic group Assosa Zone, Benishangul-Gumuz regional state, mid-west Ethiopia. *Journal of Ethnobiology and Ethnomedicine*.

[19] Alemayehu G. (2010). *Ethnobotanical study of medicinal plants used by indigenous local communities in Minjar-ShenkoraWereda, North Shewa Zone of Amhara Region, Ethiopia [M.S. thesis]*.

[20] Etana B. (2010). *Ethnobotanical study of traditional medicinal plants of Goma Wereda, Jima Zone of Oromia region, Ethiopia [M.S. thesis]*.

[21] Gebeyehu G. (2011). *An ethnobotanical study of traditional use of medicinal plants and their conservation status in Mecha Wereda, West Gojam Zone of Amhara Region, Ethiopia [M.S. thesis]*.

[22] Wabe N. T., Mohammed M. A., Raju N. J. (2011). An ethnobotanical survey of medicinal plants in the Southeast Ethiopia used in traditional medicine. *Spatula DD*.

[23] Karunamoorthi K., Tsehaye E. (2012). Ethnomedicinal knowledge, belief and self-reported practice of local inhabitants on traditional antimalarial plants and phytotherapy. *Journal of Ethnopharmacology*.

[24] Agize M., Demissew S., Asfaw Z. (2012). Ethnobotany of medicinal plants in Loma and Gena Bosa districts (woredas) of Dawro Zone, Southern Ethiopia. *Topclass Journal of Herbal Medicine*.

[25] Megersa M., Asfaw Z., Kelbessa E., Beyene A., Woldeab B. (2013). An ethnobotanical study of medicinal plants in Wayu Tuka District, East Welega Zone of Oromia Regional State, West Ethiopia. *Journal of Ethnobiology and Ethnomedicine*.

[26] d'Avigdor E., Wohlmuth H., Asfaw Z., Awas T. (2014). The current status of knowledge of herbal medicine and medicinal plants in Fiche, Ethiopia. *Journal of Ethnobiology and Ethnomedicine*.

[27] Belayneh A., Bussa N. F. (2014). Ethnomedicinal plants used to treat human ailments in the prehistoric place of Harla and Dengego valleys, eastern Ethiopia. *Journal of Ethnobiology and Ethnomedicine*.

[28] Gedif T., Hahn H.-J. (2003). The use of medicinal plants in self-care in rural central Ethiopia. *Journal of Ethnopharmacology*.

[29] Mesfin F., Demissew S., Teklehaymanot T. (2009). An ethnobotanical study of medicinal plants in Wonago Woreda, SNNPR, Ethiopia. *Journal of Ethnobiology and Ethnomedicine*.

[30] Lulekal E., Asfaw Z., Kelbessa E., Van Damme P. (2013). Ethnomedicinal study of plants used for human ailments in Ankober District, North Shewa Zone, Amhara Region, Ethiopia. *Journal of Ethnobiology and Ethnomedicine*.

[31] Regassa R. (2013). Assessment of indigenous knowledge of medicinal plant practice and mode of service delivery in Hawassa city, southern Ethiopia. *Journal of Medicinal Plants Research*.

[32] Abera B. (2014). Medicinal plants used in traditional medicine by Oromo people, Ghimbi District, Southwest Ethiopia. *Journal of Ethnobiology and Ethnomedicine*.

[33] Tolosa E. (2007). *Use and conservation of traditional medicinal plants by indigenous people in Gimbi Woreda, Western Wellega, Ethiopia [M.S. thesis]*.

[34] Suleman S., Alemu T. (2012). A survey on utilization of ethnomedicinal plants in Nekemte town, East Wellega (Oromia), Ethiopia. *Journal of Herbs, Spices & Medicinal Plants*.

[35] Gebrehowot M. (2010). *An ethnobotanical study of medicinal plants in Seru Wereda, Arsi Zone of Oromia Region, Ethiopia [M.S. thesis]*.

[36] Reta H. (2010). *An ethnobotanical study of useful plants of the farming site in Gozamen Wereda, East Gojjam Zone of Amhara Region, Ethiopia [M.S. thesis]*.

[37] Ragunathan M., Abay S. M. (2009). Ethnomedicinal survey of folk drugs used in Bahirdar Zuria district, Northwestern Ethiopia. *Indian Journal of Traditional Knowledge*.

[38] Kefyalew A. (2010). *Ethnobotanical study of medicinal plants in Ada'a Wereda, Eastern Shewa of Oromia Region, Ethiopia [M.S. thesis]*.

[39] Seid M. A., Aydagnehum S. G. (2013). Medicinal plants biodiversity and local healthcare management system in Chencha District, Gamo Gofa, Ethiopia. *Journal Pharmacognosy and Phytochemistry*.

[40] Trotter R. T., Logan M. H., Etkin N. L. (1986). Informants consensus: a new approach for identifying potentially effective medicinal plants. *Plants in Indigenous Medicine and Diet*.

[41] Bogale M., Petros B. (1996). Evaluation of the antimalarial activity of some Ethiopian traditional medicinal plants against *Plasmodium falciparum in vitro*. *SINET: Ethiopian Journal of Science*.

[42] Assefa A., Urga K., Guta M. (2007). *In vivo* antimalarial activities of plants used in Ethiopian traditional medicine, Delomenna, Southeast Ethiopia. *Ethiopian Journal of Health Science*.

[43] Deressa T., Mekonnen Y., Animut A. (2010). *In vivo* anti-malarial activities of *Clerodendrum myricoides*, *Dodonea angustifolia* and *Aloe debrana* against *Plasmodium berghei*. *Ethiopian Journal of Health Development*.

[44] Bantie L., Assefa S., Teklehaimanot T., Engidawork E. (2014). *In vivo* antimalarial activity of the crude leaf extract and solvent fractions of *Croton macrostachyus* Hocsht. (Euphorbiaceae) against *Plasmodium berghei* in mice. *BMC Complementary and Alternative Medicine*.

[45] Ayantunde A. A., Briejer M., Hiernaux P., Udo H. M. J., Tabo R. (2008). Botanical knowledge and its differentiation by age, gender and ethnicity in Southwestern Niger. *Human Ecology*.

[46] Lulekal E., Kelbessa E., Bekele T., Yineger H. (2008). An ethnobotanical study of medicinal plants in Mana Angetu District, southeastern Ethiopia. *Journal of Ethnobiology and Ethnomedicine*.

[47] Teklehaymanot T. (2009). Ethnobotanical study of knowledge and medicinal plants use by the people in Dek Island in Ethiopia. *Journal of Ethnopharmacology*.

[48] Giday M., Asfaw Z., Woldu Z. (2010). Ethnomedicinal study of plants used by Sheko ethnic group of Ethiopia. *Journal of Ethnopharmacology*.

[49] Uniyal S. K., Singh K. N., Jamwal P., Lal B. (2006). Traditional use of medicinal plants among the tribal communities of Chhota Bhangal, Western Himalaya. *Journal of Ethnobiology and Ethnomedicine*.

[50] Silva F. D. S., Ramos M. A., Hanazaki N., de Albuquerque U. P. (2011). Dynamics of traditional knowledge of medicinal plants in a rural community in the Brazilian semi-arid region. *Revista Brasileira de Farmacognosia*.

[51] Giday M., Asfaw Z., Elmqvist T., Woldu Z. (2003). An ethnobotanical study of medicinal plants used by the Zay people in Ethiopia. *Journal of Ethnopharmacology*.

[52] Asase A., Akwetey G. A., Achel D. G. (2010). Ethnopharmacological use of herbal remedies for the treatment of malaria in the Dangme West District of Ghana. *Journal of Ethnopharmacology*.

[53] Nguta J. M., Mbaria J. M., Gakuya D. W., Gathumbi P. K., Kiama S. G. (2010). Antimalarial herbal remedies of Msambweni, Kenya. *Journal of Ethnopharmacology*.

[54] Yineger H., Yewhalaw D. (2007). Traditional medicinal plant knowledge and use by local healers in Sekoru District, Jimma Zone, Southwestern Ethiopia. *Journal of Ethnobiology and Ethnomedicine*.

[55] Adefa M. S., Abraha T. B. (2011). Ethnobotanical survey of traditional medicinal plants in Tehuledere District, South Wollo, Ethiopia. *Journal of Medicinal Plants Research*.

[56] Giday M., Asfaw Z., Woldu Z. (2009). Medicinal plants of the Meinit ethnic group of Ethiopia: an ethnobotanical study. *Journal of Ethnopharmacology*.

[57] Koudouvo K., Karou D. S., Kokou K. (2011). An ethnobotanical study of antimalarial plants in Togo Maritime Region. *Journal of Ethnopharmacology*.

[58] Abebe D., Ayehu A. (1993). *Medicinal Plants and Enigmatic Health Practices of Northern Ethiopia*.

[59] Muthaura C. N., Rukunga G. M., Chhabra S. C., Mungai G. M., Njagi E. N. M. (2007). Traditional phytotherapy of some remedies used in treatment of malaria in Meru district of Kenya. *South African Journal of Botany*.

[60] Giday M., Ameni G. (2003). An ethnobotanical survey on plants of veterinary importance in two woredas of Southern Tigray, Northern Ethiopia. *SINET: Ethiopian Journal of Science*.

[61] Tabuti J. R. S., Lye K. A., Dhillion S. S. (2003). Traditional herbal drugs of Bulamogi, Uganda: plants, use and administration. *Journal of Ethnopharmacology*.

[62] Addis G., Baskaran R., Raju M. (2009). Effect of blanching and drying process on carotenoids composition of underutilized ethiopian (*Coccinia grandis* l. voigt) and Indian (*Trigonella foenum-graecum* l.) green leafy vegetables. *Journal of Food Processing and Preservation*.

[63] Teklay A., Abera B., Giday M. (2013). An ethnobotanical study of medicinal plants used in Kilte Awulaelo district, Tigray Region of Ethiopia. *Journal of Ethnobiology and Ethnomedicine*.

